# Approach to Developing a Core Competency Framework for Student Nurses in Saudi Arabia: Results from Delphi Technique

**DOI:** 10.3390/nursrep12010004

**Published:** 2022-01-25

**Authors:** Dena Attallah, Abd Alhadi Hasan

**Affiliations:** 1 Oncology and Palliative Care Nursing, Fakeeh College for Medical Sciences, Jeddah 23323, Saudi Arabia; dmattallah@fcms.edu.sa; 2 Psychiatric and Mental Health Nursing, Fakeeh College for Medical Sciences, Jeddah 23323, Saudi Arabia

**Keywords:** nursing, competence, competency, curriculum, practice, Kingdom of Saudi Arabia

## Abstract

Background: Competence, while firmly established as a primary conceptual framework in nursing education, continues to lack clarity and uniformity across borders and contexts. While a wealth of research has been carried out on the various dimensions of this concept, including the drafting and implementation of frameworks for nursing competence, no unifying international framework has been forthcoming. Indeed, the continued development of more localized approaches, based on geography or specialization, would appear to be the most realistic objective. It is incumbent on nurse educationalists and researchers to build on existing frameworks and develop evidence-based tested methodologies for competence assessment in localized contexts. Currently, there is a dearth of such evidence-based frameworks in the Middle East and in the Kingdom of Saudi Arabia (KSA) in particular. This study aimed to formulate and validate a competence framework for undergraduate nursing students in KSA. Results: Following documentary analysis, framework drafting and a three round Delphi process, a consensus was reached as to elements, comprising six discrete domains, to be included. The identified competence domains provide a framework to guide the implementation of a competence-based assessment and move towards a competency-based curriculum for nursing education in KSA. Conclusions: The study concluded that providing a competency-based model and expanding and standardization of competency concept in different dimensions of nursing profession is a necessity; considering that clarification of the concept of competency, the recognition of its dimensions, characteristics and the factors affecting it help in determining the criteria and standardizing the competency tools.

## 1. Introduction

It is widely considered that nurses are at the center of the health profession, with Mackintosh-Franklin [1] pointing out that nurses have a wide-ranging role; not only providing direct patient care but also supporting patients to perform the activities of daily life. Wu, Wang [2] stress that this critical role means it is essential for the nursing curriculum to equip graduates with the basic skills they need to address the challenges of the health system and to meet the particular needs of the local population. In Saudi Arabia, the healthcare system is a diverse model that combines the public and private sectors, with high-quality nursing services being an essential component of its delivery in hospitals [3]. To achieve the high standards of nursing that the complex care needs of patients today require, it is fundamental that nurses attain the minimum expected competencies. It is only when these professional competencies are achieved that nurses can provide high-quality care across all their duties.

Competency is described as an underlying characteristic of performance; it is multifaceted and difficult to measure. The fundamental meanings of competence and competency are similar in that ‘multiple attributes and ‘performance’ are frequently used inconsistently and interchangeably in nursing literature [4,5]. Broadly speaking, competence reflects a person’s cognitive approach to a task, encompassing the multiple attributes of knowledge, skills and attitudes [6], whereas competency highlights a person’s ability to perform those tasks within the defined context of professional practice.

The development and validation of competence frameworks for nurses and other health professionals are seen as essential elements in professional regulation and standardization and are facilitative of assessment and professional mobility [7]. This has given rise to the development of macro competence frameworks at the national level by professional regulators and governments [8,9,10,11,12,13] and at meso and micro level, subject and specialism specific competence frameworks in a range of contexts [14,15,16,17,18].

Traditional models of nursing education are frequently centered on disseminating knowledge with a limited focus on developing skills. A number of studies, including those by Fan, Gui [19] and Wu, Wang [2] showed that nursing curricula are outdated, while limited clinical training means that the clinical working environment and nursing education itself are facing considerable challenges. In developing countries in particular, traditional didactic teaching is a mainstay of nursing education, while task and apprentice-based models and the service role of nursing are still widely emphasized, according to [20]. This is certainly the case in the Middle East, where current nursing curricula are focused on these traditional criteria, a situation which Salem and Aboshaiqah [21] advise must be transformed if nurses are to meet the complex healthcare needs of patients today in the rapidly changing demographic landscape.

Benner, Tanner [22] argue that educators must train students to proficiently meet the standards that regulatory bodies require of healthcare professionals, and this means that nursing curricula should be upgraded on an ongoing basis to meet these needs. Meeting these standards helps to drive the evolution of the nursing profession and reinforces the perception of nurses as critical to driving advances in healthcare. According to Smith [23], there is no clear consensus on a single definition of competence or competency. For the purposes of this project, a working definition uses competence as a generic term that reflects a combination of skills, abilities, knowledge, and understanding. It also encompasses the capability of the individual to perform their role and ‘competency’ includes some unobservable attributes including judgment, values, and attitude [24]. In the nursing context, competence means reflecting what it means to be a professional in the field. Meanwhile, Fukada’s [25] useful definition of competency comprises the ability to meet agreed standards of work across a range of contexts, as well as the ability to execute such specific tasks effectively within the health workforce, while competency integrates multiple observable and unobservable components including knowledge and skills. 

Frank, Snell [26] indicate that rather than focusing on what students are expected to learn regarding traditional subject content, the International Bureau of Education’s definition of a competency-based curriculum (CBC) emphasizes the “complex outcomes” of the learning process, which means how they apply the right skills, knowledge, and attitudes. Other CBC definitions also stress the importance of students demonstrating that they have mastered the knowledge, values, skills, attitudes, and behaviors required for the particular degree, and advise that the instruction and assessment in the curriculum must seek to support this. The learner should be at the center of any CBC, which must be adaptable to the changing needs of the community that the students and the teachers serve, while Mackintosh-Franklin [1] stresses that key competences and competencies should be at the heart of these curricula, with students demonstrating their achievement of specified outcomes before progressing further. The complex nature of nursing competence and in constructing competence frameworks for use is emphasized furtherly by Weeks and colleagues [27,28]. In suggesting an integrated model for competence in nursing education, they stress the need to take more holistic view of the development of competence. This, in turn, has implications for teaching and learning practice, particularly in how the theory practice gap can be bridged using a framework that allows broader understanding of the concept [27]. 

The nature of the undergraduate nursing program at Fakeeh College for Medical Sciences includes 134 credit hours delivered over four years alongside one internship year. Students are introduced to wide range of the required skills in different clinical settings. For instance, adult health nursing, child health nursing, maternity health nursing, critical care nursing, psychiatric and mental health nursing and community health nursing. In each area, there are set of skills students must accomplish before leaving the module. While different contexts of practice and roles will require competencies specific to the client population and the practice settings, a set of core competencies is integral to nursing practice. These competencies are built on the undergraduate nurse’s education and experience in the areas of clinical expertise, research and professional leadership.

In Saudi Arabia, increasing levels of expectation among patients and the wider community in recent years about the quality of healthcare services have mandated the need for more effective human resources within the healthcare system, which [21] suggest has highlighted the need to focus on the clinical competence of nurses. This is also within the context of the new vision for healthcare and nursing as a whole in the Kingdom under the recently announced Saudi Vision 2030 [29,30,31]. A number of authors have pointed to challenges facing nursing and nursing education in Saudi Arabia. These include a traditional overreliance on a migrant nursing workforce, issues with the low perception of nursing as a career, and the lack of independent professional regulation [32,33,34]. While regulation and guidance are provided by the Saudi Commission for Health Specialties [35] and the Ministry for Health (MOH), there has been no articulation to date of an overarching scope of nursing practice or a macro framework for nursing competence [29]. This increased demand on health and nursing to meet the objective of Saudi Vision 2030 in turn requires many more training and education places to be created for nurses within the Kingdom. In keeping with the vison for Saudization, private universities, colleges and healthcare providers are being encouraged to become more prominent in this regard. It is within this context that the current study was carried out. This study aimed to formulate and validate a competence framework for undergraduate nursing students in KSA.

## 2. Methods

As a qualitative method of assessing the judgment of experts, the Delphi technique includes several rounds of intensive questionnaires, into which controlled opinion and feedback are interwoven. The face-to-face discussion meeting between 10 experts took place in February and April 2018. In the Delphi method used for this study, each round includes a response-analysis-response-feedback process, which was a more structured modification of the traditional Delphi technique where participants had the option to give their comments for each item. There were two phases to this Delphi technique. The inclusion criteria of experience were attained graduate level of education in nursing or medical education. In addition, exerts must have clinical and teaching experience. The study is reported consistently with the guideline of Conducting and Reporting of Delphi Studies

### 2.1. Phase I

**Figure 1:**
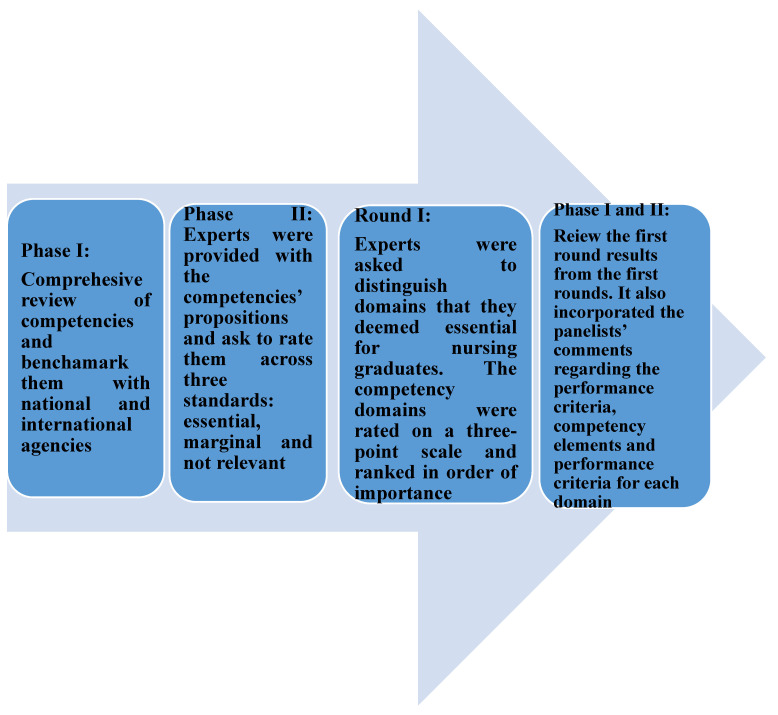
Phases of Development of Nursing professional framework.

In Phase I, a comprehensive review was conducted to determine the required competencies. The search was conducted through identification of keywords (competence, nursing, undergraduate). The following databases were used CINAHL, MEDLINE and Web of Science. Then the identified competences from the literature were benchmarked against international standards and guidelines from countries including the UK, US, Australia, Canada, and Finland. This study considered the American Association of Colleges of Nursing, the QSEN project; the UK’s Nursing and Midwifery Council (NMC); the Canadian Nursing Association’s competency framework; the Competency Outcomes and Performance Assessment (COPA) model; the US-based National League for Nursing’s (NLN) educational competencies for graduates of associate degree nursing programs, and Saudi Arabian models [8,35,36]. The derived competency framework for the essential competencies in the Bachelor of Science in Nursing (BSN) program is shown in Figure 1. There are six competencies in this framework: Person-Centered Care; Quality Care and Patient Safety; Evidence-based Practice; Professionalism and Leadership; Communication and Teamwork; Informatics and Technology.

### 2.2. Phase II

There were three rounds in Phase II, where experts were provided with the competencies’ propositions and ask to rate them across three standards: essential, marginal and not relevant. Following the completion of each round, the previous rounds’ results were embedded, with the process repeated until agreement was reached. The study was obtained the informed consent from the study participants. 

Phase II comprised the following steps:

Panel Selection: experts from the fields of nursing practice and education were contacted as potential participants as a result of their expertise and experience; they included Nurse Educators and Medical Educationists as well as Nursing Consultants. Data were collected over two months between February and April 2018, with 10 experts in total completing all three rounds of the survey.

Round I: The INC Standards of Nursing Practice and existing competency documentation were used to draft an initial competency document and in Round I, the chosen experts were asked to distinguish those domains that they deemed essential for nursing graduates. The competency domains were rated on a three-point scale and ranked in order of importance; as detailed previously, this ranged from essential through to marginal and not relevant. The experts were given the opportunity to comment on each of the domains, as well as making suggestions for modification. The panelists were not only asked to comment on the necessary knowledge and skills but also on the attitudes, values, beliefs, and behaviors that nurses need to practice effectively. The consensus criterion was set at an agreement rate of 80% and above before the Delphi rounds were started.

Round II and Round III: During these rounds, the responses from the first rounds were collated before being used to devise a revised document. Meanwhile, the new document also incorporated the panelists’ comments regarding the performance criteria, competency elements and performance criteria for each domain. The revised document was sent to the same expert panelists who had completed Round I and they were asked to use the same rating scales. The results from Round II were then collated, which enabled the document to be refined to minimize unnecessary information and remove any duplication. Round III saw the panel being asked to confirm that they agreed with the final document that had been produced in response to Round II. The process of development professional framework is described in Figure 1.

### 2.3. Ethical Considerations

The study obtained ethical approval from the Scientific Research Committee from Fakeeh College for Medical Sciences (42/2019). All study participants provided informed consent form and their confidentiality is assured.

## 3. Results

Eight out of the ten experts who agreed to take part in the Delphi study were nurses with either clinical experience, academic experience, or both, while the remaining two were medical educationists as described in Table 1. Round I resulted in six domains of core competencies being agreed upon, all of which had an initial consensus rate of 70% or over; Person-Centered Care; Quality Care and Patient Safety; Evidence-based Practice; Professionalism and Leadership; Communication and Teamwork; Informatics and Technology. To achieve the 80% consensus agreed beforehand, those experts who had not found a competency to be relevant in this round were sent the results for feedback and 80% consensus was subsequently achieved for all six domains, as described in Table 2. To achieve the 80% consensus agreed beforehand, those experts who had not found a relevant competency in this round were given the results for feedback, then the 80% consensus was subsequently achieved for all six domains, as described in Table 2.

**Figure 2:**
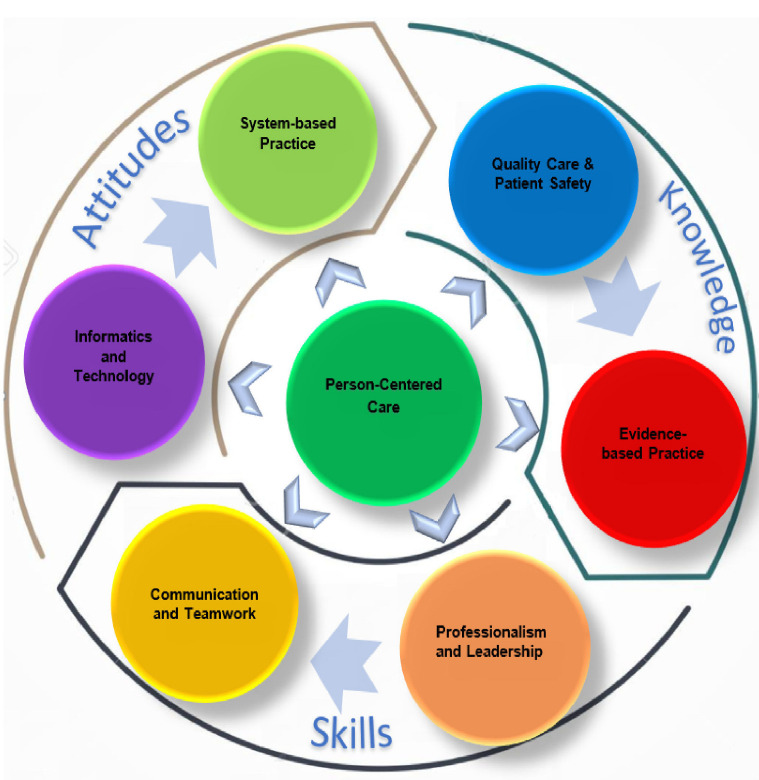
Core Competency Framework for Undergraduate Nursing Student.

Table 2 shows the results of Round II, most of the items resulted in an 80% consensus. The final Core Competency Framework draft comprised 16 competency elements across six competency domains, and each of the competency elements included a description of all the required performance behavior, as reported in Table 3 and Figure 2.

Table 2 shows the results of Round II. Most of the items resulted in an 80% consensus. The final Core Competency Framework draft comprised 16 competency elements across six competency domains, each of the competency elements included a description of all the required performance behavior, as reported in Table 3 and Figure 2.

## 4. Discussion

The iterative process described in this paper results in the production of the competence framework for BSc nursing as demonstrated in Figure 2. The framework draws on many different sources and narratives, both from the international literature and local guidelines and governance structures, for nursing in KSA. Following the rounds of the Delphi process described above, the 6-domain framework provides a foundational basis for the development and assessment of student nurse competence in KSA. The key strength of this framework is that it marries the cultural and local requirements of undergraduate nursing in KSA with existing and established frameworks from other parts of the world.

The employment of established frameworks to be adopted in local contexts is a previously used method by other authors [37,38]. Operating on the premise that a broad framework for nursing student competence should be internationally transferable would appear to be useful [39]. Batt, Tavares [7] do however caution that this can lead to the replication of frameworks that may not in themselves have solid foundations in the first place. In the context of the development of nursing and Saudization in KSA [29], it is an important starting point to use frameworks from elsewhere.

The use of the Delphi process to develop a framework for competence in nursing and other health professions is commonplace and offers a structured technique to obtain expert review and consensus [38,40]. Critics of the methodology suggest that the robustness of the process is dependent on having actual experts and being clear about consensus means [41]. In the current study, we predefined the number of rounds that would be included in the Delphi process [3] and ensured a mix of clinical and academic expertise in our panel with particular reference to expertise in nursing in KSA. As suggested by Diamond, Grant [42] these steps enhance the trustworthiness of the process.

The size and the composition of the expert panel may limit the generalizability of the study. A panel size of fifteen fits within the Delphi guidelines as reliability is shown to be maximized with a panel size of 10, though it remains unclear if a larger sample size and a panel of nursing experts from different parts of the world would have altered the level of agreements on the competency domains.

## 5. Conclusions

This study developed the core competency framework for the undergraduate nursing students using a Delphi technique and identified six competency domains in the areas of person-centered care, professionalism and leadership, evidence-based practice, communication and teamwork, information and technology. This competency framework will provide regulatory bodies with a preliminary draft to work on a large scale and keeping national interests in mind to help in the credentialing/licensing procedure. This will allow the regulatory bodies to explore the assessment process for the ‘fitness of practice’ of new graduates.

## Figures and Tables

**Table 1:** Sociodemographic Data of the Study Expert.

	Frequency	Percent
Education Qualification		
PhD	9	90%
Master	1	10%
Clinical experience		
<5 years	2	20%
6–10 years	3	30%
>10 years	5	50%
Teaching experience		
<5 years	1	10%
6–10 years	4	40%
>10 years	5	50%
Speciality		
Nursing	8	80%
Non-Nursing	2	20%

**Table 2:** Core Competency Domains and the ordering of the Domains after Round 1.

	Source	Initial	Ordering after Round	Agreement
Person-Centered Care	QSEN/ANMC/NMC	1	1	8/10
Quality Care and Patient Safety	ANMC/AACN/COPA	6	3	9/10
Evidence-based Practice	AACN/NMC	4	4	9/10
Professionalism and Leadership	AACN/NMC/CP/OPA	2	2	10/10
Communication and Teamwork	AACN/NMC	3	5	9/10
Informatics and Technology	AACN/QSEN/COPA	5	6	8/10

**Table 3:** Competency domains and competency elements after Round III.

Core Competency Framework	Source	Consensus
1. Person-Centered Care	QSEN/ANMC/NMC	
1.1. Holistic care		90%
1.2. Partner with patient in care delivery		100%
1.3. Health promotion		90%
1.4. Pain and symptom management		80%
2. Quality Care and Patient Safety	ANMC/AACN/COPA	
2.1. Manage risk and promote safety		100%
3. Evidence-based Practice	AACN/NMC	
3.1. Clinical decision- making		80%
3.2. Reflective clinical practice		80%
4. Professionalism and Leadership	AACN/NMC/CP/OPA	
4.1. Accountability		100%
4.2. Change-agent		90%
4.3. Management of patient care		80%
5. Communication and Teamwork	AACN/NMC	
5.1. Professional interaction		100%
5.2. Nurture shared decision-making		80%
5.3. Foster mutual respect		90%
6. Informatics and Technology	AACN/QSEN/COPA	
6.1 Use informatics		80%
7. System-based Practice	QSEN/ANMC/NMC	
7.1. System effectiveness		80%
7.2. System efficiency		80%

## Data Availability

Data are available upon the request.

## References

[1] Mackintosh-Franklin C. (2016). Nursing philosophy: A review of current pre registration curricula in the UK. Nurse Educ. Today.

[2] Wu F.Q., Wang Y.L., Wu Y., Guo M. (2014). Application of nursing core competency standard education in the training of nursing undergraduates. Int. J. Nurs. Sci..

[3] Landeen J., Carr D., Culver K., Martin L., Matthew-Maich N., Noesgaard C., Beney-Gadsby L. (2016). The impact of curricular changes on BSCN students’ clinical learning outcomes. Nurse Educ. Pract..

[4] Leung K., Trevena L., Waters D. (2016). Development of a competency framework for evidence-based practice in nursing. Nurse Educ. Today.

[5] Laibhen-Parkes N. (2014). Evidence-based practice competence: A concept analysis. Int. J. Nurs. Knowl..

[6] Saunders H., Vehviläinen-Julkunen K. (2018). Key considerations for selecting instruments when evaluating healthcare professionals’ evidence-based practice competencies: A discussion paper. J. Adv. Nurs..

[7] Batt A.M., Tavares W., Williams B. (2020). The development of competency frameworks in healthcare professions: A scoping review. Adv. Health Sci. Educ..

[8] NMC (2014). Standards for Competence for Registered Nurses.

[9] American Association of Colleges of Nursing (2012). Graduate-Level QSEN Competencies: Knowledge, Skills and Attitudes. https://www.aacnnursing.org/Portals/42/AcademicNursing/CurriculumGuidelines/Graduate-QSEN-Competencies.pdf.

[10] Nursing and Midwifery Board of Ireland (2016). Nurse Registration Programmes Standards and Requirements.

[11] Massachusetts Department of Higher Education Nursing (2016). Massachusetts Nurse of the Future Nursing Core Competencies.

[12] Canadian Nurses Association C. (2015). Framework for the Practice of Registered Nurses in Canada.

[13] National League for Nursing N. (2010). Outcomes and Competencies for Graduates of Practical/Vocational, Diploma, Associate Degree, Baccalaureate, Master’s, Practice Doctorate, and Research Doctorate Programs in Nursing.

[14] Aggar C., Gordon C.J., Thomas T.H.T., Wadsworth L., Bloomfield J. (2018). Evaluation of a community transition to professional practice program for graduate registered nurses in Australia. Nurse Educ. Pract..

[15] Attard J., Ross L., Weeks K.W. (2019). Design and development of a spiritual care competency framework for pre-registration nurses and midwives: A modified Delphi study. Nurse Educ. Pract..

[16] Lopez-Medina I.M., Álvarez-Nieto C., Grose J., Elsbernd A., Huss N., Huynen M., Richardson J. (2019). Competencies on environmental health and pedagogical approaches in the nursing curriculum: A systematic review of the literature. Nurse Educ. Pract..

[17] Kim S.-O., Choi Y.-J. (2019). Nursing competency and educational needs for clinical practice of Korean nurses. Nurse Educ. Pract..

[18] Díaz Agea J.L., Martín Robles M.R., Jiménez Rodríguez D., Morales Moreno I., Viedma Viedma I., Leal Costa C. (2018). Discovering mental models and frames in learning of nursing ethics through simulations. Nurse Educ. Pract..

[19] Fan L., Gui L., Xi S., Qiao A. (2016). Core competence evaluation standards for emergency nurse specialist: Developing and testing psychometric properties. Int. J. Nurs. Sci..

[20] Brownie S.M., Docherty C., Al-Yateem N., Gadallah M.H., Rossiter R. (2018). Developing a national competency-based curriculum for technical nurses in Egypt. East. Mediterr. Health J..

[21] Salem O.A., Aboshaiqah A.E., Mubaraki M.A., Pandaan I.N. (2018). Competency Based Nursing Curriculum: Establishing the Standards for Nursing Competencies in Higher Education. Open Access Libr. J..

[22] Benner P.E., Tanner C.A., Chesla C.A. (2009). Expertise in Nursing Practice: Caring, Clinical Judgment, and Ethics.

[23] Smith S.A. (2012). Nurse competence: A concept analysis. Int. J. Nurs. Knowl..

[24] Hemalatha R., Shakuntala B. (2018). A Delphi Approach to Developing a Core Competency Framework for Registered Nurses in Karnataka, India. Nitte Univ. J. Health Sci..

[25] Fukada M. (2018). Nursing competency: Definition, structure and development. Yonago Acta Med..

[26] Frank J.R., Snell L.S., Cate O.T., Holmboe E.S., Carraccio C., Swing S.R., Harris P., Glasgow N.J., Campbell C., Dath D. (2010). Competency-based medical education: Theory to practice. Med. Teach..

[27] Weeks K.W., Coben D., O’Neill D., Jones A., Weeks A., Brown M., Pontin D. (2019). Developing and integrating nursing competence through authentic technology-enhanced clinical simulation education: Pedagogies for reconceptualising the theory-practice gap. Nurse Educ. Pract..

[28] Weeks K.W., Pontin D. (2020). Modelling the landscape of professional nursing competence—A global perspective. Nurse Educ. Pract..

[29] AL-Dossary R.N. (2018). The Saudi Arabian 2030 vision and the nursing profession: The way forward. Int. Nurs. Rev..

[30] Alsufyani A.M., Alforihidi M.A., Almalki K.E., Aljuaid S.M., Alamri A.A., Alghamdi M.S. (2020). Linking the Saudi Arabian 2030 vision with nursing transformation in Saudi Arabia: Roadmap for nursing policies and strategies. Int. J. Afr. Nurs. Sci..

[31] Kingdom of Saudi Arabia Vision 2030. https://vision2030.gov.sa/en.

[32] Anthony D., Alosaimi D., Dyson S., Korsah K.A., Saleh M. (2020). Development of nurse education in Saudi Arabia, Jordan and Ghana: From undergraduate to doctoral programmes. Nurse Educ. Pract..

[33] Almalki M., FitzGerald G., Clark M. (2011). The nursing profession in Saudi Arabia: An overview. Int. Nurs. Rev..

[34] AlYami M., Watson R. (2014). An overview of nursing in Saudi Arabia. J. Health Spec..

[35] Saudi Commission for Health Specialties (2014). Guideline of Professional Classification and Registration for Health Practitioners.

[36] Albarqouni L., Hoffmann T., Straus S., Olsen N.R., Young T., Ilic D., Shaneyfelt T., Haynes R.B., Guyatt G., Glasziou P. (2018). Core competencies in evidence-based practice for health professionals: Consensus statement based on a systematic review and Delphi survey. JAMA Netw. Open.

[37] Gill F.J., Leslie G.D., Grech C., Latour J.M. (2015). An analysis of Australian graduate critical care nurse education. Collegian.

[38] Zhang X., Meng K., Chen S. (2020). Competency framework for specialist critical care nurses: A modified Delphi study. Nurs. Crit. Care.

[39] Torres-Alzate H. (2019). Nursing Global Health Competencies Framework. Nurs. Educ. Perspect..

[40] Galbraith K., Ward A., Heneghan C. (2017). A real-world approach to Evidence-Based Medicine in general practice: A competency framework derived from a systematic review and Delphi process. BMC Med. Educ..

[41] Lock L.R. (2011). Selecting examinable nursing core competencies: A Delphi project. Int. Nurs. Rev..

[42] Diamond I.R., Grant R.C., Feldman B.M., Pencharz P.B., Ling S.C., Moore A.M., Wales P.W. (2014). Defining consensus: A systematic review recommends methodologic criteria for reporting of Delphi studies. J. Clin. Epidemiol..

